# Improving walking speed reduces hospitalization costs in outpatients with cardiovascular disease. An analysis based on a multistrata non-parametric test

**DOI:** 10.1186/s12913-020-05874-3

**Published:** 2020-11-17

**Authors:** Stefano Bonnini, Gianni Mazzoni, Michela Borghesi, Giorgio Chiaranda, Jonathan Myers, Simona Mandini, Andrea Raisi, Sabrina Masotti, Giovanni Grazzi

**Affiliations:** 1 Department of Economics and Management, University of Ferrara, Ferrara, Italy; 2 Center for Exercise Science and Sport, University of Ferrara, Via Gramicia , 35, 44121 Ferrara, Italy; 3 Public Health Department, AUSL Ferrara, Ferrara, Italy; 4 Center for Modelling Computing and Statistics, University of Ferrara, Ferrara, Italy; 5 Public Health Department, AUSL Piacenza, Piacenza, Italy; 6 General Directorship for Public Health and Integration Policy, Emilia-Romagna Region, Bologna, Italy; 7 Division of Cardiology, VA Palo Alto, Palo Alto, CA USA; 8 Stanford University School of Medicine, Stanford, CA USA

**Keywords:** Cardiovascular disease, Secondary prevention, Physical activity, Hospitalization costs, Permutation test

## Abstract

**Background:**

To assess the association between walking speed (WS) and its improvement on hospitalization rates and costs in outpatients with cardiovascular disease.

**Methods:**

Six hundred forty-nine patients participating in an exercise-based secondary prevention program were studied. Patients were divided at baseline into two groups characterized by low and high WS based on the average WS maintained during a moderate 1-km treadmill-walking test. WS and other covariates were grouped into three domains (demographic factors, medical history and risk factors), and used to estimate a propensity score, in order to create homogeneous groups of patients. All-cause hospitalization was assessed 3 years after baseline as a function of WS. Hospitalization and related costs were also assessed during the fourth-to-sixth years after enrollment. To test whether the hospitalization costs were related to changes in WS after 36 months, a multistrata permutation test was performed by combining within strata partial tests.

**Results:**

The results support the hypothesis that hospitalization costs are significantly reduced in accordance with an improvement in WS. This effect is most evident among older patients, overweight or obese, smokers, and those without a history of coronary artery bypass surgery.

**Conclusions:**

The present study supports growing evidence of an inverse association between WS, risk of hospitalization and consequent health-care costs. The joint use of propensity score and multistrata permutation approaches represent a flexible and robust testing method which avoids the possible effects of several confounding factors typical of these studies.

**Supplementary Information:**

The online version contains supplementary material available at 10.1186/s12913-020-05874-3.

## Background

Cardiovascular disease (CVD) is the leading cause of death globally, and is associated with an immense health and economic burden [1, 2]. Prioritization of health behaviors (including physically active lifestyle), in addition to the treatment of established CVD, is a primary goal of numerous leading organizations worldwide to improve cardiovascular health and reduce healthcare costs [2, 3]. Despite this overwhelming evidence, a considerable percentage of world’s population has an insufficient level of physical activity (PA) [4–6], making it one of the most prevalent major CVD risk factors [7–9]. Recent estimates indicate that ~ 5.3 million deaths per year are directly attributable to insufficient PA, similar to smoking-related mortality globally [7, 10]. In addition, inactivity imposes a large economic burden, recently quantified as ~$120 billion and ~ € 80 billion per year in the US and in the European Union, respectively [11, 12], mostly attributable to hospital costs [13]. Cost-effectiveness and feasibility of PA promotion in current clinical practice is well supported [7, 14–16]. This growing evidence and policy support has led to a number of alternative and pragmatic approaches to deliver proven and sustainable models that can be integrated into modern healthcare settings [7].

One key approach to increase PA and health across the population is to promote walking for recreation and transport [17–19]. Walking is the most common type of leisure-time PA among adults, and is simple, cost-effective, and with a strong potential to improve public health [20, 21]. Walking speed (WS), measured as the time required to walk a given distance, is a common tool used to assess physical function [22]. WS has also been demonstrated to be inversely associated with disability, hospitalization, and survival in patients with CVD [23–26]. Indeed, WS has been suggested as the ‘sixth vital sign’ [27], and its clinical assessment has been recommended along with heart rate, breathing, temperature and pain [27]. WS determination requires low resources, time, and skills, making it suitable for routine assessment in clinical and research settings [22]. Slow walking speed (WS) has been associated with disability and higher risk for hospitalization and mortality in patients with CVD [23–26].

Evidence for the prognostic value of WS is largely based on a single measure at baseline. However, since PA habits can change during a given follow-up period, inferences based on a single measure at baseline could lead to erroneous conclusions [28]. The current study was conducted to investigate whether WS is associated with all-cause hospitalization costs, and whether serial changes in WS are accompanied by changes in long-term hospitalization costs. The joint application of propensity scoring and multistrata permutation represents a flexible and robust testing method, suitable in the presence of confounding factors typical of these studies.

## Methods

### Study population

Six hundred forty-nine consecutive patients were enrolled in an exercise-based secondary prevention program at the Center for Exercise and Sports Science at the University of Ferrara, Italy, and Department of Public Health, AUSL Ferrara, Italy. Patients were referred by their GP or cardiologist from at least 3 months up to several months after discharge. Patients were clinically stable based on the absence of need to modify overall therapy for at least 3 months before baseline examination, and with no symptoms at rest and during light to moderate exercise intensities during daily activities. The goal of the program was to promote long-term physically active lifestyles in outpatients with CVD. An unsupervised home program consisting of 30–60 min of brisk walking, at least 3–4 days per week, was recommended. Before admission, participants underwent a comprehensive clinical evaluation, including medical history. The study was approved by the Human Studies Committee of the University of Ferrara, no. 22–13, and all subjects gave written informed consent.

### Follow-up and hospitalization

Patients were evaluated between October 1997 and January 2013. Follow-up sessions were created to motivate patients to promote and maintain physically active lifestyles. Follow-up booster visits were created to encourage patients and stimulate further involvement in physically active lifestyle. History of changes in PA or symptoms, and assessment of walking capacity were assessed at any visit. During these booster sessions, the subject’s individualized goals (i.e. achievement of recommended level of PA) were discussed and were used as a basis for adjustment of the exercise prescription. Benefits associated with improved exercise capacity were explained, emphasizing the health consequences of being able to walk faster and longer.

On admission, quarterly during the first 2–3 years, and thereafter twice per year, each patient performed a 1-km treadmill-walking test at moderate perceived exercise intensity, at a value of 11–13/20 on the 6–20 Borg scale, as previously described (1 k-TWT) [29].

Benefits associated with improved WS were explained, emphasizing the health consequences of being able to walk faster and longer. Participants were flagged by the regional Health Service Registry of the Emilia-Romagna Region, which provided data on hospitalization. Any admission was considered an event. For patients experiencing > 1 hospitalization, only the first event was considered in the analysis.

### Data analysis

Several covariates were considered to account for possible confounding effects. Baseline WS reflects the general physical condition of subjects at a certain time [30], and may affect the potential of subjects to improve their WS. By using the median as a threshold value, patients were divided into two groups characterized by low and high WS at the baseline. The resulting dichotomous variable represented one covariate in the analysis. The other covariates were grouped into three domains: demographic factors, medical history and risk factors. At baseline, patient’s CVD diagnosis was defined from the hospital discharge record. Blood chemistry analyses previously performed and left ventricular ejection fraction derived from a prior echocardiographic evaluation were registered. The demographic factors were age and gender variation 36 months after baseline. The medical history was represented by four dummy variables. Each variable indicates the possible presence of a specific event in a given patient’s medical history. The events considered were myocardial infarction, coronary artery bypass graft (CABG), percutaneous transluminal coronary angioplasty (PTCA) and valvular repair/replacement. The risk factors considered were glycaemia and five dummy variables indicating the presence of established risk factors (hypertension, angina, family history, hypercholesterolemia), or unhealthy behaviors (smoking status). To test whether the cost reduction increased as a function of the WS variation and vice-versa, the patients were classified according to whether the WS variation was high (greater than the median) or low (less than or equal to the median), and the two groups were compared in terms of mean cost variation.

Supplemental Table 1 shows the mean values and standard deviations of the hospitalization cost and of the covariates for each of the two groups.

### The model

Let the random variable *C*_*it*_ represent the hospitalization cost of patient *i* in the three-year period *t*, with *t = 1,2*. The outcome *Y*_*i*_ *= C*_*i2*_ *- C*_*i1*_ represents the cost variation for the i-th patient and it is the response variable of the study. The sample of *n* patients can be bipartite into two subgroups *H* and *L*, depending on whether the increase in the WS walking speed after 36 months was high or low respectively. We tested the hypothesis of a significant negative effect of the WS increase on the cost variation. The problem can be represented as follows:

1 $$ {Y}_i={\mu}_L+{\delta}_H{w}_i+{\varepsilon}_i $$

where *ε*_*i*_ is a zero-mean homoscedastic random error with *Var*(*ε*_*i*_) = *σ*^2^, *μ*_*L*_ = *E*(*Y*_*i*_| *i* ∈ *L*) is the mean cost variation for the group of patients with low WS increase, *w*_*i*_ is the dummy variable that represents the membership to group *H* and *δ*_*H*_ is the effect of the WS increase on the cost variation.

*The testing problem.*

The hypotheses of the problem are:

2 $$ {H}_0:{\delta}_H=0 $$

and

3 $$ {H}_1:{\delta}_H<0. $$

In the null hypothesis *H*_0_, there is no effect of the WS increase because *μ*_*H*_ = *E*(*Y*_*i*_| *i* ∈ *H*) = *μ*_*L*_ + *δ*_*H*_ = *μ*_*L*_, and in the alternative hypothesis *H*_1_ there is a negative effect thus *μ*_*H*_ < *μ*_*L*_.

To take into account the possible confounding effects of covariates, we classified the patients into homogeneous strata according to the confounding factors, performed two-sample within-stratum tests and combined them.

### The nonparametric method

A methodological solution can be found in the family of combined permutation tests [31–34], suitable for several complex testing problems [35, 36]. The hypotheses (2) and (3) can be broken down into k > 1 partial null hypotheses and k > 1 partial alternative hypotheses. The k partial tests are combined to solve the global test.

The *p*-values of the partial and global tests are computed according to the null permutation distribution of the test statistics (under the assumption of exchangeability in the null hypothesis). Without loss of generality, let us assume that the j-th partial null hypothesis is rejected for large values of the test statistic *T*_*j*_ and *L*_*j*_(*x*) = *Pr* [*T*_*j*_ ≥ *x*] be the significance level function. Hence, by denoting with ${T}_j^{(b)}$ the value of the j-the partial test statistic corresponding to the b-th permutation of the dataset, with *b* = 1, …, *B*, and with ${T}_j^{(0)}$ the observed value of the test statistic, the *p*-value of the j-th partial test is

$$ {l}_j={L}_j\left({T}_j^{(0)}\right)={\sum}_{b\ge 1}I\left({T}_j^{(b)}\ge {T}_j^{(0)}\right)/B, $$

where *B* is the total number of permutations and *I*(*x*) is the indicator function that takes value 1 when *x* is true. For computational convenience, a random sample of the set of permutations (draws with replacement) can be used and estimated *p*-values can be computed instead of the exact ones, ${\hat{l}}_j=\left[{\sum}_{b\ge 1}I\left({T}_j^{(b)}\ge {T}_j^{(0)}\right)+0.5\right]/\left(B+1\right)$. The value of the combined test statistic corresponding to the b-th permutation is ${T}_{comb}^{(b)}=\psi \left[{L}_1\left({T}_1^{(b)}\right),\dots, {L}_k\left({T}_k^{(b)}\right)\right]$ and the estimated *p*-value of the global test is ${\hat{l}}_{comb}=\hat{L}\left({T}_{comb}^{(0)}\right)$. The combination function *ψ* must be non increasing in the arguments and must attain its supremum (strictly greater than the significance level *α*) when at least one argument tends to zero.

In case of significance of the global test, it may be of interest to know which partial tests determine the global significance. However, a suitable control of the familywise error rate (probability of wrong rejection of one partial null hypothesis) must be applied, to avoid that the probability of type I error (wrong rejection of the global *H*_0_) may exceed the significance level *α*. Thus the significance of the *j*-th partial test must be assessed by comparing its *p*-value with an adjusted (reduced) significance level or, equivalently, the adjusted partial *p*-value ${}_{adg}{\hat{l}}_j$ (greater than or equal to the original partial *p*-value ${\hat{l}}_j$) with the significance level *α* [37]. A powerful solution is the so called Bonferroni-Holm procedure [38], which is preferable to the conservative Bonferroni rule where ${}_{adg}{\hat{l}}_j=k{\hat{l}}_j$. According to the Bonferroni-Holm procedure

4 $$ {}_{adg}{\hat{l}}_{(j)}={\max}_{r\le j}\left\{\min \left[1,\left(k-r+1\right){\hat{l}}_{(j)}\right]\right\} $$

where ${\hat{l}}_{(j)}$ denotes the j-th ordered estimated partial *p*-value. Given that we are considering a multiple test with the goal of determining contribution of the partial tests to the possible global significance by computing the Bonferroni-Holm adjustment of the *p*-values, the Tippett function seems to be the suitable choice for the tests combination.

### The propensity score approach for the stratification

One of the main confounding factors is the WS at the baseline and, since we are interested to assess its possible confounding effect, it is considered separately with respect to the other confounders. All the other factors are too many to be considered separately for the stratification purpose. Thus the propensity score technique is applied [39, 40]. Let us assume that the membership to H of patient *i* follows a Bernoulli distribution with parameter *π*_*i*_ and that the relationship between *π*_*i*_ and the confounders can be represented by the logit model log[*π*_*i*_/(1 − *π*_*i*_)] = *β*_0_ + ∑_*j*_*β*_*j*_*x*_*ij*_ + *ξ*_*i*_, where *x*_*ij*_ is the observed value of the *j*-th confounding factor on patient *i*, *ξ*_*i*_ is the random error of the model and *β*_0_, ⋯, *β*_*k*_ are unknown parameters. The estimated propensity score is

5 $$ {\hat{\pi}}_i=\mathit{\exp}\left({\hat{\beta}}_0+{\sum}_j{\hat{\beta}}_j{x}_{ij}\right)/\left[1+\mathit{\exp}\left({\hat{\beta}}_0+{\sum}_j{\hat{\beta}}_j{x}_{ij}\right)\right] $$

Where ${\hat{\beta}}_0,\cdots, {\hat{\beta}}_k$ are suitable estimates of *β*_0_, ⋯, *β*_*k*_ respectively.

To define homogeneous strata of patients, according to the confounding factors, the estimated propensity score quantiles can be used as thresholds to determine the membership to strata. For example, the tertiles can be used to split data into three groups that represent homogeneous strata of patients.

### Multistrata test

Patients were initially divided into two groups based on WS performance during the 1 k-TWT at baseline, and classified as fast walkers or slow walkers for WS values above and below the median value, respectively. Next, patients were classified into low and high improvers according to variation in WS 3 years after baseline. To assess the association between WS, WS variation and incidence of 3 year hospitalization, we constructed Kaplan-Meier curves. Significantly correlated variables were entered into a fully adjusted Cox regression model, with first hospitalization as the outcome.

By jointly considering (i) the bipartition of the patients according to the median of the WS at the baseline (1:low, 2:high) and (ii) the tripartition of the patients according to the tertiles of the estimated propensity score based on the other confounding factors (1:low, 2:medium, 3:high), six different strata are determined and then the test on the effect of the WS increase on the cost variation can be broken down into six partial tests.

The null hypothesis of the problem can be represented as follows

6 $$ {H}_0:{\cap}_v{\cap}_s{H}_{0, vs} $$

where *v* = 1, 2 denotes the group according to criterion (i) and *s* = 1, 2, 3 the group according to criterion (ii), and *H*_0, *vs*_ is the partial null hypothesis of no effect within stratum (*v*, *s*). *H*_0_ is true if and only if all the partial null hypotheses are true. Similarly, the alternative hypothesis can be formally written as

7 $$ {H}_1:{\cup}_v{\cup}_s{H}_{1, vs} $$

Where *H*_1, *vs*_ is the partial alternative hypothesis of significant (negative) effect within stratum (*v*, *s*). *H*_1_ is true when at least one partial alternative hypothesis is true.

For the random errors *ε*_1_, ⋯, *ε*_*n*_ defined in (1), the usual independence assumption typical of the parametric tests is not necessary. Since a permutation approach is applied, the weaker condition of exchangeability is assumed.

In order to perform the propensity score analysis, all the covariates of direct interest (age, sex, BMI, AMI, CABG, PTCA, Valve repair/replacement, hypertension, total cholesterol, creatinine levels, fasting glucose, smoking habit, and medications) and the information about the belonging of each patient to one of the two groups based on the walking speed variation, were included in a Minitab software worksheet. By means of a binary logistic regression, *P*-values of every covariate were investigated in order to determine their significance, whose level was set at *P* ≤ 0.1. Regression equation coefficients were also estimated in order to evaluate the effect of each covariate on the probability that they are linked to WS variation. As said, baseline WS was considered separately. We then tested the hypothesis that hospitalization costs, within each stratum and overall, were lower for the individuals classified as High improvers compared to those classified as Low improvers running the multistrata permutation test with the software NPC Test. The significance level was set at the 0.05. Those who were never admitted to hospital were considered as cost zero (€ = 0).

## Results

### Baseline walking speed and 3-year hospitalization

**Fig. 1:**
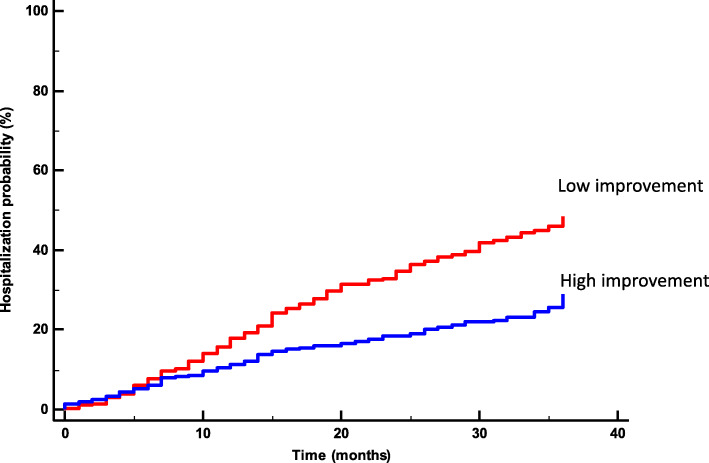
Rate of hospitalization during 36 months after enrolment stratified according to walking speed at baseline

Baseline characteristics of the participants are presented in Table 1. The median WS at baseline was 4.2 km/h. During the 3 years after baseline, 227 subjects (35% of the sample) were hospitalized with an incidence of 43 and 27% for slow and fast walkers, respectively. The cumulative risk of hospitalization by groups of WS is presented in Fig. 1 (log rank, *P* < 0.0001). In the logistic binary regression, only age, BMI variation, history of CABG and smoking status affected the probability of being in the fast walkers group. In the presence of history of CABG, the probability was higher, while the influence of age, BMI increase and being a current smoker were not significant. Cox analysis showed that, compared to the slowest group, the HR for hospitalization was lower in the fastest group (HR 0.85, 95%CI 0.73–0.99, *P* = 0.038). After adjustments for confounders, every 1 km/hour increase in WS was associated with a 21% reduction in risk of hospitalization (HR 0.79, 95% CI 0.71–0.89, *P* < 0.001).

**Table 1:** Characteristics of the subjects at the baseline by groups of walking speed

	All subjects	SLOW walkers	FAST walkers
	(***n*** = 649	(***n*** = 332)	(***n*** = 317)
**Walking speed (km/h)**	4,2 (1,0)	3,4 (0,7)	5,0 (0,6)
**General**			
Age (yr)	63 (9)	66 (9)	60 (9)
Gender (M/F)	568/81	263/69	305/12
BMI (kg/m^2^)	27,9 (3,8)	28,4 (4,0)	27,4 (3,5)
LV ejection fraction (%)	56 (10)	55 (10)	58 (9)
**Risk factor**			
Current smoking (%)	5	3	6
Hypertension (%)	60	70	55
Famili history (%)	53	44	62
Fasting glucose (mg/dL)	106 (25)	108 (26)	104 (23)
Total cholesterol (mg/dL)	194 (41)	196 (41)	191 (42)
HDL cholesterol (mg/dL)	50 (12)	50 (12)	50 (12)
Serum truglycerides (mg (dL)	135 (67)	137 (37)	132 (68)
Serum creatinine (mg/dL)	1,09 (0,3)	1,13 (0,3)	1,05 (0,2)
**Medical history**			
CABG (%)	56	60	52
Myocardial infarction (%)	21	18	27
PTCA (%)	8	6	9
Valvular repair/replacement (%)	11	13	8
Other (%)	4	3	4
**Medications**			
ACE inhibitor or ARB (%)	55	60	50
Aspirin (%)	75	73	77
β-blockers (%)	59	56	62
Calcium antagonists (%)	13	14	12
Diuretics (%)	15	24	5
Statins (%)	55	49	61

Values are presented as mean (standard deviation, SD) or %. *Abbreviations*: *ACE* Angiotensin-Converting Enzyme, *ARB* Angiotensin Receptor Blocker, *BMI* Body Mass Index, *CABG* Coronary Artery Bypass Graft, *HDL* High-density lipoproteins, *LV* Left Ventricular, *PTCA* Percutaneous Transluminal Coronary Angioplasty, stenting or both

### Walking speed improvement and 4 to 6 years’ hospitalization

**Fig. 2:**
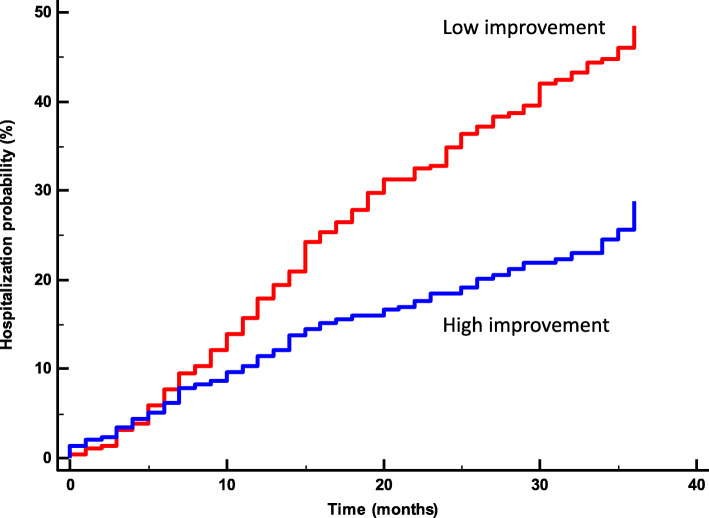
Rate of hospitalization 36 to 72 months after enrolment stratified according to walking speed improvement

WS among the entire study population improved 3 years after baseline from 4.2 ± 1.0 to 5.0 ± 1.1 km/hour. The improvements in WS in the slow and fast groups at baseline were 3.4 ± 0.7 to 4.3 ± 1.0 km/hour, and 5.0 ± 0.6 to 5.7 ± 0.7 Km/hour, respectively. During 4-to-6 years after baseline, 211 subjects (36% of the sample) were hospitalized. Figure 2 shows the Kaplan-Meier survival curves for hospitalization by groups of WS improvement. The incidences of hospitalization were 44 and 28% for Low and High improvers (log rank *P* < 0.0001). Among the interactions between WS change and covariates, only age, gender, BMI variation, family history of CVD, and history of CABG were statistically significant. The lower hospitalization rate for WS improvement persisted after adjustment for confounders. Cox regression analysis showed that, compared to the slowest group, the HR for hospitalization was lower in the fastest group (HR 0.51, 95%CI 0.38–0.68, *P* = 0.0001). Every 1 km/hour increase in WS was associated with an adjusted 39% reduction in risk for hospitalization (HR 0.61, 95%CI 0.49–0.76, *P* < 0.0001).

The logistic binary regression, to study the relationship between the propensity score and covariates related to demographic information, medical history and risk factors, was performed with a stepwise method to determine the significance of the coefficients. At the significance level *α* = 0.05, only age, BMI variation, CABG and smoking affect the probability to be in group H (high increase of WS). In the presence of CABG, the mentioned probability is higher; instead, the effect on the mentioned probability caused by age, BMI increase and being a smoker is negative. The application of (5) allows to estimate the propensity score of each patient as function of age, BMI variation, CABG and smoking (Table 2).

**Table 2:** Propensity score analysis: binary logistic regression for dummy high speed increase (dependent variable)

Explanatory variable	Estimated coefficient	***p***-value	Odds Ratio
Constant	22,053	0,000	–
**Demographic covariates**			
Age	−0,0383	0,000	0,96
Gender (1:Male; 0:Female)	n.s.		
Body Mass Index Variation	−0,2465	0,000	0,78
**Medical history**			
Myocardial infarction	n.s.		
Coronary artery bypass	0,4988	0,003	1,65
Percutaneous transluminal coronary angioplasty	n.s.		
Valvular replacement	n.s.		
**Risk factors**			
Glycemia	n.s.		
Smoke	-0,7870	0,048	0,46
Hypertension	n.s.		
Angina	n.s.		
Familiar history	n.s.		
Hypercholesterolemia	n.s.		

*n.s.* Non significant

a = 0.05

The first tertile of the estimated propensity scores is 0.44, the second tertile is 0.55. The median of the WS at the baseline resulted 4.2 km/h. In Table 3 we can see that, according to the combined multi-strata permutation test, the hypothesis of no effect must be rejected at *α* = 0.05 in favor of the hypothesis that an increase of WS lowers the hospitalization costs (global *P*-value = 0.020).

**Table 3:** Multistrata permutation test on the effect of walking speed increase on hospitalization cost

Average walking speed at baseline	Propensity score	Sample size	***P***-value	Adjusted ***P***-value
Low	Low	124	0,003	0,020
	Medium	110	0,640	0,955
	High	98	0,673	0,955
High	Low	90	0,005	0,023
	Medium	115	0,785	0,955
	High	112	0,283	0,738
	Global Test	649	0,020	

According to the adjusted *P*-values, the global significance must be attributed to the sub-groups “low WS at the baseline and low propensity score” (stratum 1) and “high WS at the baseline and low propensity score” (stratum 4).

As reported in Table 4, stratum 1 consists of patients with a mean age equal to 70 (oldest group among the three with low WS at baseline), a mean BMI increase of 1.37 (highest difference among the three groups with low WS at the baseline), a 5% of smokers, and 38% with CABG (maximum and minimum respectively, when the WS at the baseline is low). Among the patients with high WS at baseline, stratum 4 is the oldest (mean age 64), the one with highest BMI increase (1.20 mean variation), with the lowest percentage of patients with CABG (29%) and the highest percentage of smokers (19%). Thus, the effect of WS increase on the cost reduction is significant in old patients with great BMI increase. The absence of CABG facilitates the significant effect of the WS increase on the costs, which is relevant among the smokers. Hospitalization costs in the first, second and third propensity score tertile per patient were reduced from 1131 to 301 €, from 798 to 338 €, and from 1057 to 295 € among low and high improvers, respectively.

**Table 4:** Strata characterization in terms of rilevant confounding factors

Average walking speed at baseline	Propensity score	Mean age	Mean BMI var.	Coronary art. bypass (%)	Smoke (%)
Low	Low	69,66	1,37	37,90	4,84
	Medium	66,96	0,03	72,73	2,73
	High	59,01	−1,02	72,45	2,04
High	Low	64,41	1,20	28,89	18,89
	Medium	61,35	0,43	53,91	1,74
	High	54,03	−0,27	67,86	0,89

## Discussion

In this cohort of outpatients with CVD referred to an exercise-based secondary prevention program, slower WS at baseline was associated with a higher risk of all-cause hospitalization over 1 to 3 years. These results further support the inverse relationship between walking capacity and long-term risk of morbidity and mortality in outpatients with ischemic heart disease [41, 42], and chronic heart failure [43], as well as in those undergoing cardiac surgery [24]. The second notable finding was the reduced rates and costs of hospitalization associated with the improvement in WS between 4 and 6 years after baseline. After propensity score analysis and through permutation testing we observed a significant cost reduction for the high improvers compared to the low improvers. In particular, the significance of the hospitalization cost reduction as a consequence of an increase in WS can be attributed to the oldest patients, those with high BMI, the absence of coronary artery bypass surgery, and smokers. We have empirical evidence that this is true for both the WS levels at the baseline.

These findings are in agreement with previous data demonstrating that similar PA interventions may account for between € 1120 and € 15,860 per quality-adjusted life-year gained, which can be more cost-effective than drug-based interventions [7, 43]. These results further support growing evidence related to reduced health care costs in more active individuals [13, 44]. The findings of this study extend to a broad spectrum of individuals with a wide range of age (28–88 years), WS (1.2–7.1 km/h), and demographic and clinical characteristics.

Several factors likely contribute to explain these results. Physical activity has an important role in the prevention and management of > 40 chronic conditions.7 However, while established CVD risk factors are assessed routinely in clinical practice [45, 46], PA is commonly not assessed [47]. In this study we assessed WS, which reflects the combined functioning of the cardiorespiratory, nervous and musculoskeletal systems [48, 49]. The moderate WS determined by the endurance treadmill-walking protocol employed in this study was inversely associated with survival in a large cohort of outpatients with CVD [50]. Favorable associations between WS improvement and hospitalization rate and costs resulted independent from age and clinical history. In addition, we jointly utilized two different but complementary statistical methods - combined permutation and propensity score – to investigate differences in hospitalization rates and costs.

Another relevant aspect of this study concerns the utilization of serial measurements over a long follow-up. The prognostic value of WS in ageing and chronic diseases is well documented. However, this evidence is mostly based on a unique assessment at baseline. Since lifestyles can change over a given follow-up period, there is a great deal of value in assessing WS serially [28].

### Implications for policy

This study further supports the use of exercise-based secondary prevention programs in a broad range of patients with CVD [47]. Traditional interventions in patients with CVD consist primarily of supervised exercise sessions delivered in a hospital [51]. However, notable evidence indicates that these models are often neither financially viable nor sustainable [52, 53]. Thus, creating and implementing alternative approaches have been advocated [5]. Home-based models have been shown to be effective and safe [54–56], and are less time consuming and logistically easier for many patients [5, 57, 58]. These models, similar to the one presented herein, are an option for those patients who cannot easily access hospital- or facility-based programs [58].

### Strengths of the study

First, there was a large sample size of patients across a wide range of age and functional capacity. Second, the walking test protocol is simple, and a potential easy tool to apply in clinical practice. Furthermore, walking is the most frequently performed form of physical activity [59, 60], and the preferred activity by insufficiently active individuals, regardless of age and sex [61]. Yet, the incorporation of goals meaningful to a given patient for performing activities of daily living such moderate WS has been recommended [53]. Fourth, the non-restrictive inclusion criteria employed is likely to reflect real-world clinical practice. Fifth, the study was based on an analysis of administrative data and used an analytic matching approach and a multistrata test to control for potential confounding. This permitted us to assess specific characteristics of the patient subgroups for which the effect of walking speed variation was significant, increasing the validity of findings. Finally, to our knowledge, no other study has examined the effects of improvement in WS on hospitalization costs in cardiac outpatients during a follow-up of this length.

### Limitations of the study

First, our results may not apply to different settings, which may have different policies for health services use and costs. Second, this approach does not fully adjust for the severity of cardiovascular risk profile or comorbidity [62–64], including injury-related hospitalization. Third, this study was carried out in patients who chose to participate in an exercise-based secondary prevention program and thus, they may not be representative of the general population with similar cardiovascular conditions. Fourth, given the modest number of women, the findings of this study should be used with caution among females. Fifth, adherence to the recommended home program was not determined, and thus, a causal relationship between the “dose” of PA and the WS improvement is not possible. However, we recently demonstrated in a randomized controlled trial in older outpatients with CVD that the intervention produced significant increase in habitual PA, associated with improved health outcomes [65–67].

## Conclusions

The present study contributes to growing evidence that supports the inverse association between slow WS and risk of hospitalization, and lower health-care costs. These results provide evidence that moderate WS is a simple, potentially useful, and clinically important indicator of health for outpatients with CVD. The test may help health professionals identify less fit patients which may require greater use of health services, ultimately requiring greater monetary expenditures. Finally, the findings may help health professionals seeking a simple method to reduce the prevalence of sedentary behavior that remains extremely high among patients with CVD.

## Supplementary Information

**Additional file 1: Table S1.** Hospitalization cost variation and covariates (mean ± SD) versus walking speed variation (dichotomized).

## Data Availability

The dataset generated and analysed during the current study are not publicly available due to limits determined by the rules of our health system in terms of sensitive data management. However, data are available from the corresponding author on reasonable request.

## Abbreviations

BMI: Body Mass Index
WS: Walking speed
CVD: Cardiovascular disease
PA: Physical activity
1 k-TWT: 1-km treadmill-walking test
CABG: Coronary artery bypass graft
PTCA: Percutaneous transluminal coronary angioplasty stenting or both
ACE: Angiotensin-Converting Enzyme
ARB: Angiotensin Receptor Blocker
HDL: High-density lipoproteins
LV: Left Ventricular
