# Supplementary material for: Improving walking speed reduces hospitalization costs in outpatients with cardiovascular disease. An analysis based on a multistrata non-parametric test
Source: BMC Health Serv Res. 2020 Nov 17;20:1048. doi: 10.1186/s12913-020-05874-3 (PMC7670683; doi:10.1186/s12913-020-05874-3)
Supplement: Supplementary file 1 — Additional file 1: Table S1. Hospitalization cost variation and covariates (mean ± SD) versus walking speed variation (dichotomized). [file 12913_2020_5874_MOESM1_ESM.docx]

**Supplemental Table 1 - Hospitalization cost variation and covariates (mean ± SD) versus walking speed variation (dichotomized).**

| **Variable** | **Walking speed variation** | | | | | | **Difference** |
| --- | --- | --- | --- | --- | --- | --- | --- |
|  |  | **low (£0.80)** |  |  | **high (>0.80)** |  | **(high - low)** |
|  |  |  |  |  |  |  |  |
| Cost variation | 837,0166 | ± | 7359,7138 | -208,2126 | ± | 9944,6734 | -1045,2293 |
|  |  |  |  |  |  |  |  |
| **Covariates** |  |  |  |  |  |  |  |
|  |  |  |  |  |  |  |  |
| Baseline walking speed | 4,3863 | ± | 1,0908 | 3,9508 | ± | 0,9156 | -0,4354 |
|  |  |  |  |  |  |  |  |
| Age | 63,8663 | ± | 8,9970 | 61,4938 | ± | 8,9796 | -2,3725 |
| Gender (1:Male; 0:Female) | 0,8571 | ± | 0,3505 | 0,8938 | ± | 0,3086 | 0,0366 |
| Body Mass Index Variation | 0,5310 | ± | 1,4778 | 0,0775 | ± | 1,4833 | -0,4535 |
|  |  |  |  |  |  |  |  |
| Myocardial infarction | 0,2158 | ± | 0,4120 | 0,1969 | ± | 0,3983 | -0,0189 |
| Coronary artery bypass | 0,5198 | ± | 0,5004 | 0,5969 | ± | 0,4913 | 0,0771 |
| Percutaneous transluminal coronary angioplasty | 0,0973 | ± | 0,2968 | 0,0531 | ± | 0,2246 | -0,0441 |
| Valvular replacement | 0,1094 | ± | 0,3126 | 0,1000 | ± | 0,3005 | -0,0094 |
|  |  |  |  |  |  |  |  |
| Glycemia | 106,9483 | ± | 26,9603 | 105,4150 | ± | 22,0782 | -1,5333 |
| Smoke | 0,0608 | ± | 0,2393 | 0,0344 | ± | 0,1825 | -0,0264 |
| Hypertension | 0,6322 | ± | 0,4829 | 0,5594 | ± | 0,4972 | -0,0728 |
| Angina | 0,7234 | ± | 0,4480 | 0,7031 | ± | 0,4576 | -0,0203 |
| Familiar history | 0,4954 | ± | 0,5007 | 0,5656 | ± | 0,4965 | 0,0702 |
| Hypercholesterolemia | 0,0760 | ± | 0,2654 | 0,0875 | ± | 0,2830 | 0,0115 |
